# Case report: Total thoracoscopic repair of sinus of Valsalva aneurysm combined with ventricular septal defect

**DOI:** 10.3389/fcvm.2022.1023501

**Published:** 2022-10-21

**Authors:** Tong Tan, Haozhong Liu, Jianrui Ma, Jian Liu, Haiyun Yuan, Huiming Guo

**Affiliations:** ^1^Department of Cardiovascular Surgery, Guangdong Provincial Key Laboratory of South China Structural Heart Disease, Guangdong Cardiovascular Institute, Guangdong Provincial People’s Hospital, Guangdong Academy of Medical Sciences, Guangzhou, China; ^2^Shantou University Medical College, Shantou, China

**Keywords:** Valsalva aneurysm, ventricular septal defect, total thoracoscopic surgery, transaortic approach, congenital heart surgery

## Abstract

The sinus of Valsalva aneurysm (SVA) is a rare cardiac anomaly. It can develop into the heart failure if it ruptures, which requires early intervention. However, such congenital anomalies are usually treated using a median sternotomy approach. Here, we report a rare case of SVA combined with a ventricular septal defect in which the patient underwent patch repair of the defects under a total thoracoscopy approach. She was discharged uneventfully and showed no residual shunt or aortic regurgitation postoperatively or at the 12-month follow-up. The total thoracoscopic approach for SVA repair is technically feasible.

## Introduction

A sinus of Valsalva aneurysm (SVA) is an abnormal bulge located between the aortic valve annulus and the sinotubular junction that does not belong to any coronary sinuses. It is a rare cardiac anomaly occurring in less than 1% of the cases (1). It is predominant in males (4:1) and most common in young Asian individuals (2, 3). The natural history of SVA has been suggested to contribute to a deficiency of the elastic fibers between the aortic media and the annulus fibrosis layer (4). The sinus of origin most commonly involves the right coronary, followed by the non-coronary and the left coronary. Most unruptured SVAs are asymptomatic, depending on the degree of left to right shunt and associated lesions, but SVA rupturing into the ventricular system may cause acute onset symptoms and even heart failure in severe cases. Since the first successful surgery of SVA was reported by Lillehei et al. (5), the safety and efficiency of thoracotomy surgery to repair SVA has been demonstrated in numerous studies (2, 5, 6). We performed repair of the shunts under total thoracoscopy for a patient diagnosed with SVA combined with the ventricular septal defect (VSD).

## Case presentation

**FIGURE 1:**
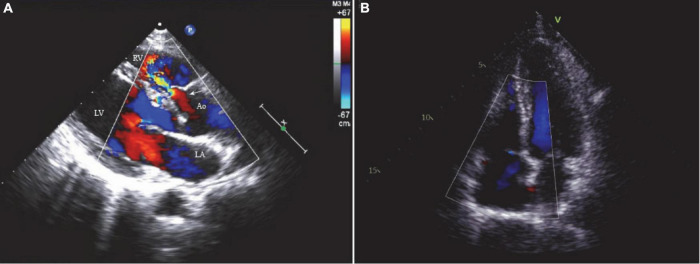
**(A)** The two-dimensional echocardiogram showed a 10.6 mm × 9.6 mm ruptured sinus of Valsalva aneurysm (right coronary sinus) and a 5.0 mm aortocardiac shunt (*white arrow*). **(B)** No residual shunt or valve regurgitation noted on the postoperative echocardiogram. LA, left atrium; LV, left ventricle; RV, right ventricle; Ao, aorta.

A 48-year-old female visited our clinic with complaints of dyspnea for 4 months. On arrival, her vital signs were stable, including a body temperature of 36.1°C, a heart rate of 96 beats/min, a respiratory rate of 20 breaths/min, and a blood pressure of 121/67 mmHg. Jugular vein distension or limb edema was absent. A continuous third-degree heart murmur was noted over the left sternal border. She was diagnosed with SVA at the age of 15. However, she was asymptomatic until 6 years ago, when she experienced occasional shortness of breath and fatigue. Surgery was recommended, but she accepted medical therapy for fear of cardiac surgery. Four months prior to her presentation, her symptoms were exacerbated, and she developed dyspnea. Transthoracic echocardiography (TTE) showed a 10.6 mm × 9.6 mm dilated SVA with a ruptured right coronary sinus into the right ventricular outflow tract (RVOT) (Figure 1A). The diameter of the aortocardial shunt was approximately 5.0 mm, and turbulent blood flow was observed from the aorta to the RVOT with a peak flow velocity of 4.9 m/s and a pressure gradient of 96 mmHg. The aortic regurgitation was traced, but the aortic valve anatomy and function were normal. The atria and ventricles remained normal in size. The ejection fraction of the left ventricle was 64%. No defects involving the atrial septum or the ventricular septum, endocarditis, or severe valvular regurgitation were noted on TTE. However, transesophageal echocardiography (TEE) revealed a small infundibular VSD with no other congenital pathology.

**FIGURE 2:**
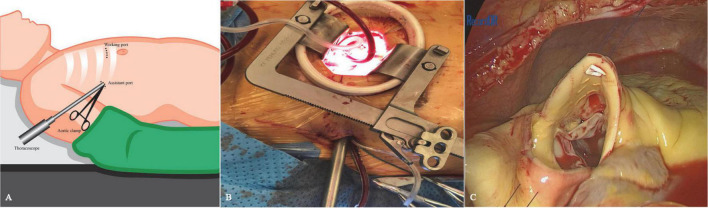
Schematic diagram of the thoracoscopic procedure. **(A)** The patient was positioned in a supine position with the right chest elevated by 15 degrees. The working port was placed at the third intercostal space on the mid-clavicular line, while the thoracoscopic port was made in the third intercostal space at the level of the anterior axillary line. **(B)** Thoracoscopic approach view. **(C)** To fully expose the aortic root, two or three retraction sutures were placed over the transected aorta.

The patient provided written and fully informed consent, and she underwent surgical repair through a totally thoracoscopic approach. After insertion of a double-lumen tracheal intubation under general anesthesia, the patient was placed in the supine position with the right chest elevated by 15 degrees (Figure 2A). Cannulation will be carried out through the femoral artery and vein. A soft tissue retractor was inserted into the working port established in the third intercostal space lateral to the right midclavicular line (Figure 2B). The assistant port was made in the third intercostal space in the anterior axillary line, where the thoracoscope, an aortic cross-clamp and a left ventricle vent catheter were placed (Figure 2A). Cardiopulmonary bypass and moderate systemic hypothermia were initiated. The ascending aorta was cross-clamped and then transected. Antegrade perfusion of histidine–tryptophan–ketoglutarate cardioplegia solution was infused directly into the coronary ostia. In total, two or three retraction sutures were placed over the transected aorta to achieve full exposure of the aortic root (Figure 2C). An infundibular VSD was detected with its superior border connected to the right coronary valve of the aortic valve. The SVA originating in the right coronary sinus was also found, from which an aorto-right ventricular fistula was created. The VSD and the SVA were repaired with two separate bovine pericardial patches with interrupted 4–0 monofilament sutures and reinforced at the edge with Gore-Tex pledgetted sutures (Supplementary Video 1). The effect of patch repair was evaluated by saline injection, and postoperative TEE confirmed that no interventricular or aorto-right ventricular shunt flow existed. The cardiopulmonary bypass time (CPB) was 127 min, and the aortic cross-clamp (ACC) time was 88 min. The patient stayed in the ICU for 1 day and was discharged uneventfully 5 days postoperatively. At the 12-month follow-up, TTE demonstrated no residual shunt or valve regurgitation (Figure 1B).

## Discussion

The SVA can be either ruptured or non-ruptured. Non-ruptured SVA is usually asymptomatic, while ruptured SVA presents with symptoms of ventricular overload, such as dyspnea and decreased activity tolerance. The symptoms of ruptured SVA might be delayed due to good tolerance of the right heart. Patients with ruptured SVAs usually express themselves at an average age of 34 (ranging from 11 to 67 years old) (7). For ruptured SVAs, Sakakibara and Konno first proposed a classification system to distinguish their site of origin and rupture (8). However, it only includes SVAs originating from the right coronary sinus (Types I to III), non-coronary (Type IV) and protruding into the right ventricle and right atrium. Xin-Jin et al. proposed a modified Sakakibara classification system with five types that is simpler and more practical for clinical use, according to the site of SVA rupture (9).

Although SVA is rare, it is frequently associated with other congenital cardiac anomalies, such as VSD, aortic regurgitation, and tetralogy of Fallot (10–12). The combined VSD could be a membranous, perimembranous, supracristal or muscular type, and the supracristal type is the principal type associated with ruptured SVA (13). However, VSD could be missed preoperatively by TTE, as in our case. Some case reports contributed to the aortocardiac shunt overlapping with the VSD shunt, which might also occur in our case. Other reasons included either the large aneurysmal sacs embedded into the VSD or the shunt flow across the VSD reduced by high right ventricular pressure (14–16). Therefore, TEE, three-D echocardiogram, CT or MRI are recommended modalities to improve the early detection rate of VSD and the accuracy of diagnosis (16, 17).

**FIGURE 3:**
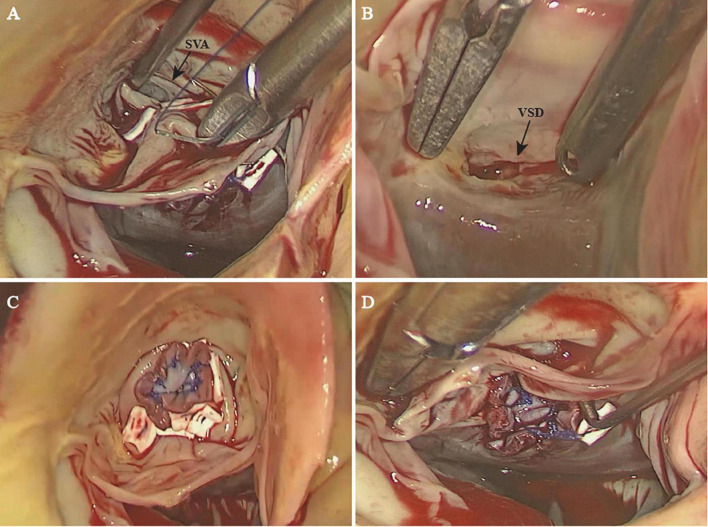
The sinus of Valsalva with a ruptured right coronary sinus **(A)** and the ventricular septal defect **(B)** were exposed well through the endoscope. The sinus of Valsalva aneurysm **(C)** and the ventricular septal defect **(D)** were successfully repaired with a patch. SVA, sinus of Valsalva aneurysm; VSD, ventricular septal defect.

Ruptured SVAs require early surgical repair, and excellent long-term outcomes have been reported (18). Apart from the traditional open surgical approach, including aortotomy, right atriotomy, and right ventriculotomy, the minimally invasive surgical approach has become increasingly preferred. In our case, the patient was satisfied with the minimal invasiveness and cosmetic results of total thoracoscopy. In addition, the incidence of SVA is higher in young to middle-aged adults; the total thoracoscopic approach has a better chance of reoperation than median sternotomy. The total thoracoscopic technique has been reported and effectively used for some congenital cardiac diseases, such as VSD and atrial septal defect (19, 20). Fukumoto et al. (21) reported a case of aorto-right ventricular fistula using 3D endoscopy. To the best of our knowledge, our case is the first reported case of total thoracoscopic repair of SVA combined with VSD. Our approach was similar to the thoracoscopic aortic valve replacement at our center. The endoscope provides a good view of the right coronary cusp and non-coronary cusp while exposing the infundibular VSD well (Figures 3A,B). In some cases, the SVA was associated with cardiovascular lesions, fistula opening to the right atrium, and even severe RVOT obstruction (22–24). Note such complex cardiac anomalies require more detailed surgical exploration and more manipulation instead of simple repair with patch through transaortic approach. We carefully evaluated preoperative imaging data (especially TEE) and applied this approach for excellent exposure of the aneurysm and the VSD that were easily targeted. Such an approach is technically feasible, as we successfully repaired both the SVA and VSD with patches without residual shunts or aortic regurgitation noted postoperatively (Figures 3C,D) or at the 12-month follow-up. The CPB and ACC times were comparable to those of the conventional approach, and our patient made an expedient recovery and was discharged uneventfully. We also have extensive experience with aortic valve surgery; therefore, aortic valvuloplasty or aortic valve replacement could be performed under endoscopy if necessary.

In conclusion, total thoracoscopic repair of selected SVAs combined with VSD is a minimally invasive procedure with a clear visual field of the orifice of the fistula. Such an approach is technically feasible and has excellent outcomes.

## Data availability statement

The original contributions presented in the study are included in the article/Supplementary material, further inquiries can be directed to the corresponding author.

## Ethics statement

This study was performed in accordance with the Declaration of Helsinki and approved by the Ethics Committee of Guangdong Provincial People’s Hospital, Guangdong Academy of Medical Sciences (No. KY-Q-2022-223-01). The patients/participants provided their written informed consent to participate in this study. Written informed consent was obtained from the individual(s) for the publication of any potentially identifiable images or data included in this article.

## Author contributions

TT and HL contributed to study design, data acquisition, and manuscript drafting. JM, JL, HY, and HG contributed greatly to the revision of the manuscript. HG approved the submission of the final version. All authors contributed to the article and approved the submitted version.
